# Dactylolysis spontanea (ainhum)

**DOI:** 10.1590/0100-3984.2014.0064

**Published:** 2015

**Authors:** Ronaldo Garcia Rondina, Ricardo Andrade Fernandes de Mello, Gabriel Antônio de Oliveira, Laís Bastos Pessanha, Luiz Felipe Alves Guerra, Diego Lima Nava Martins

**Affiliations:** 1 Universidade Federal do Espírito Santo, Vitória, ES, Brazil.

**Figure 1:**
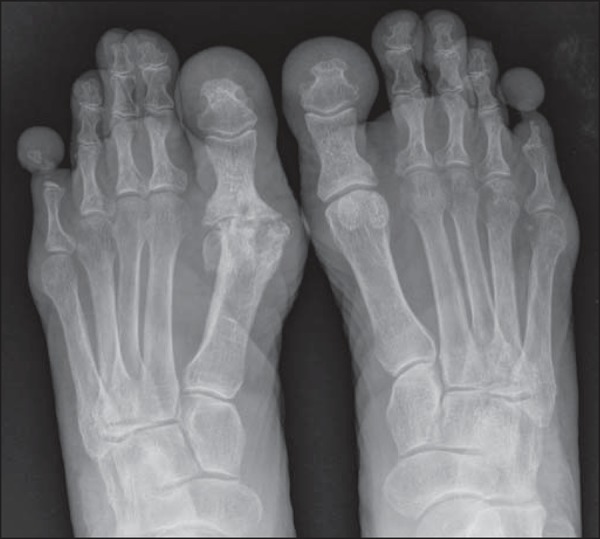
Radiography – anteroposterior view of the feet two years after the symptoms onset. In addition to the narrowing and osteolysis of the fifth middle and distal phalanges, particularly at left, incidental degenerative diseases are observed in the metatarsal phalangeal joint of the right hallux.

*Dear Editor*,

A 76-year-old, white woman presenting with bone resorption in the fifth toes. For three years, the patient had experienced severe pain and local edema. Conventional radiography (Figure 1) demonstrated narrowing and osteolysis of the middle and distal phalanges of the fifth toes, most noticeable at left, in association with focal and concentric decrease in the soft parts thickness at the roots of those toes. Because of the intense local pain, the patient underwent surgical amputation of the fifth toes, and the symptoms disappeared.

Several spontaneously painful conditions of lower and upper limbs, particularly in their extremities, have been observed and reported in Brazil^(1-5)^.

Dactylolysis spontanea (DS), that is also known as “ainhum”, is a rare disease occurring principally in Afrodescendant male individuals (2:1) aged between 30 and 50^(6)^. “Ainhum” is an Angolan word meaning “to saw”. The first case report of DS in Brazil refers to a “quilombola” in Bahia state and was described by Silva Lima in 1867^(^7^)^. The prevalence of DS ranges from 0.015 to 2% of the population in African countries. In Brazil, its prevalence is still to be studied.

Few reports of cases of DS in white individuals are found in the literature^(8)^. In Brazil, due to ethnic miscegenation, a higher incidence of this disease may be observed in white individuals with some African ancestry that is not always noticeable in their phenotype.

The main DS feature of this condition is the development of a fibrotic constriction ring involving the base of one or more toes, conditioning eversion and absorption of distal structures, possibly progressing to spontaneous amputation^(9)^. Recently a case of DS involving toes and fingers was reported^(9)^.

The radiographic findings are typical and may be classified into four phases, as follows: the first one is characterized by the development of a deep sulcus along the medial aspect of the distal portion of the proximal phalanx, sometimes resembling the shape of a sand glass. The second phase progresses with increase in the volume distally to the constriction ring, secondary to lymphedema. The third phase is characterized by progressive bone absorption, and the fourth phase, by spontaneous amputation averagely occurring within fourth to six years after the disease onset^(9)^.

The differential diagnosis should be made with other conditions involving the development of fibrotic constriction rings such as porokeratosis of Mibelli; erythropoietic protoporphyria; scleroderma; psoriasis; neuropathic plica; hanseniasis; syphilis; Raynaud’s disease; diabetes mellitus and syringomyelia. Also, facticious pseudoainhum, caused by hair-thread tourniquet syndrome should be considered^(9)^.

No well established treatment for DS is available yet. Resection of the sulcus followed by z-plasty may alleviate the pain and avoid spontaneous amputation at early stages of the disease^(6)^. Surgical amputation may be recommended to alleviate the symptoms.^(10)^

DS is a rare disease whose diagnosis is hampered by its low prevalence and variable clinical presentation. The radiological evaluation allows for an early diagnosis, preventing spontaneous amputation.
